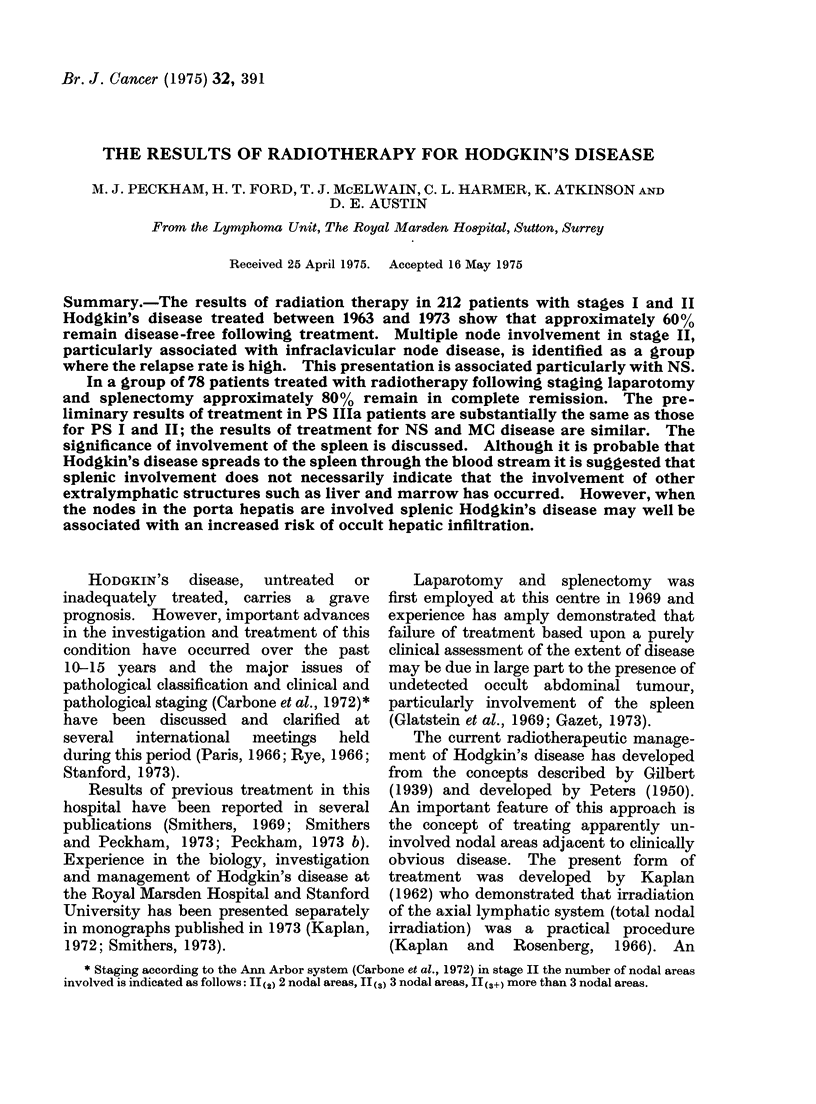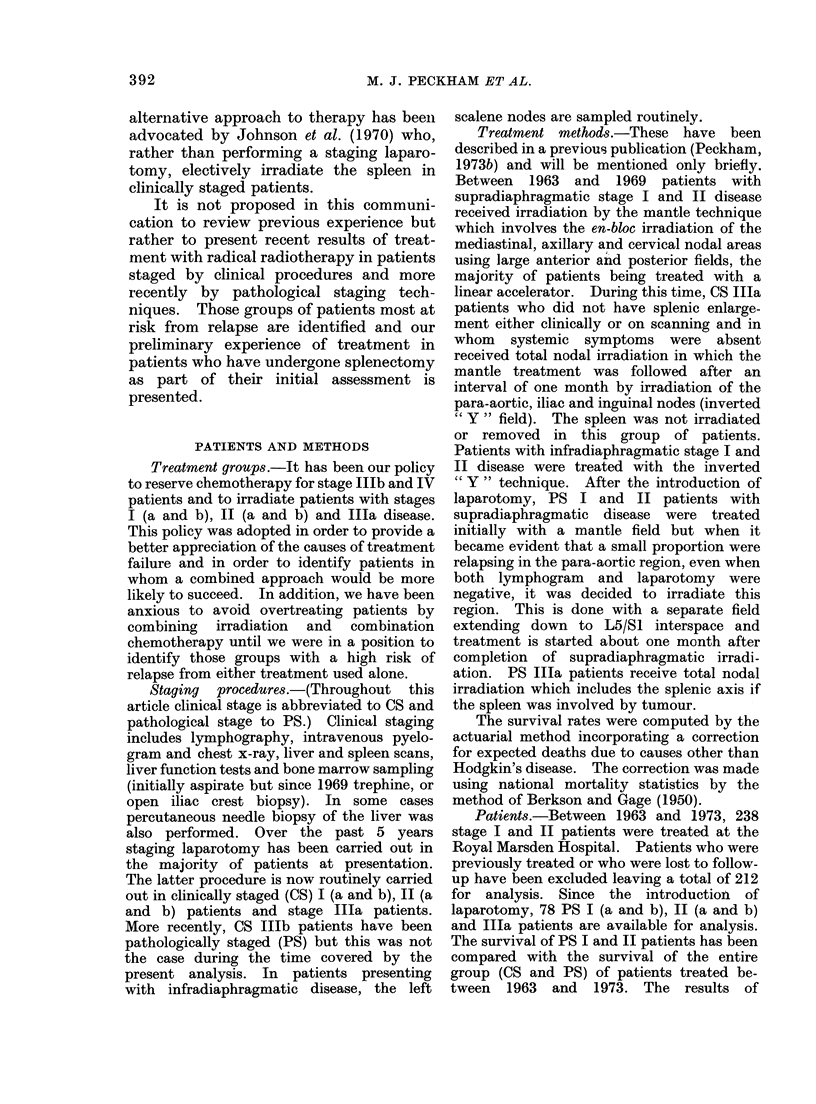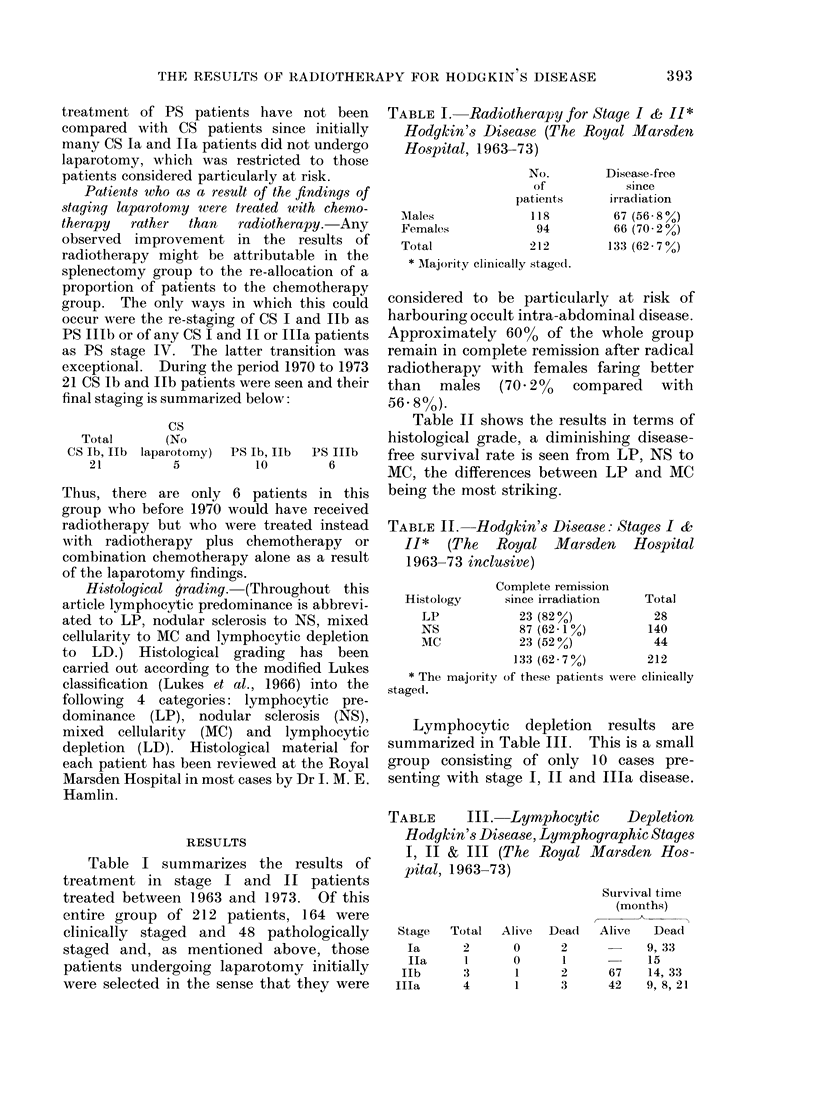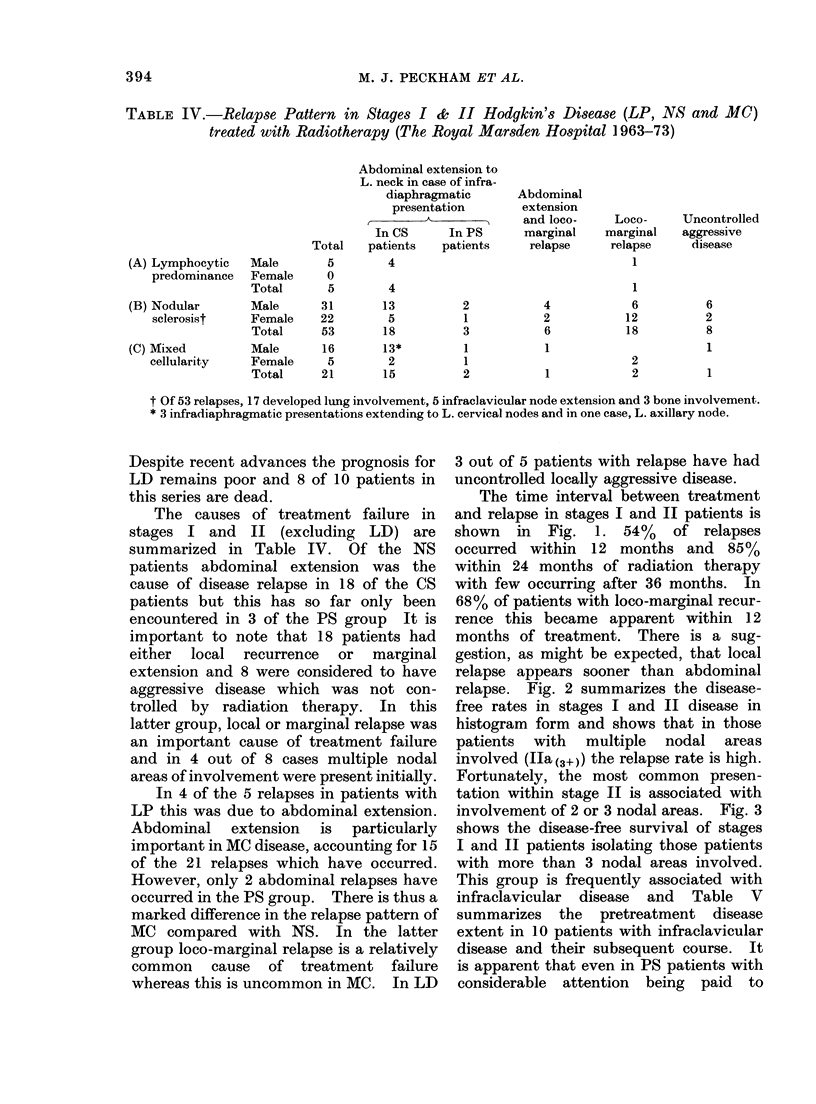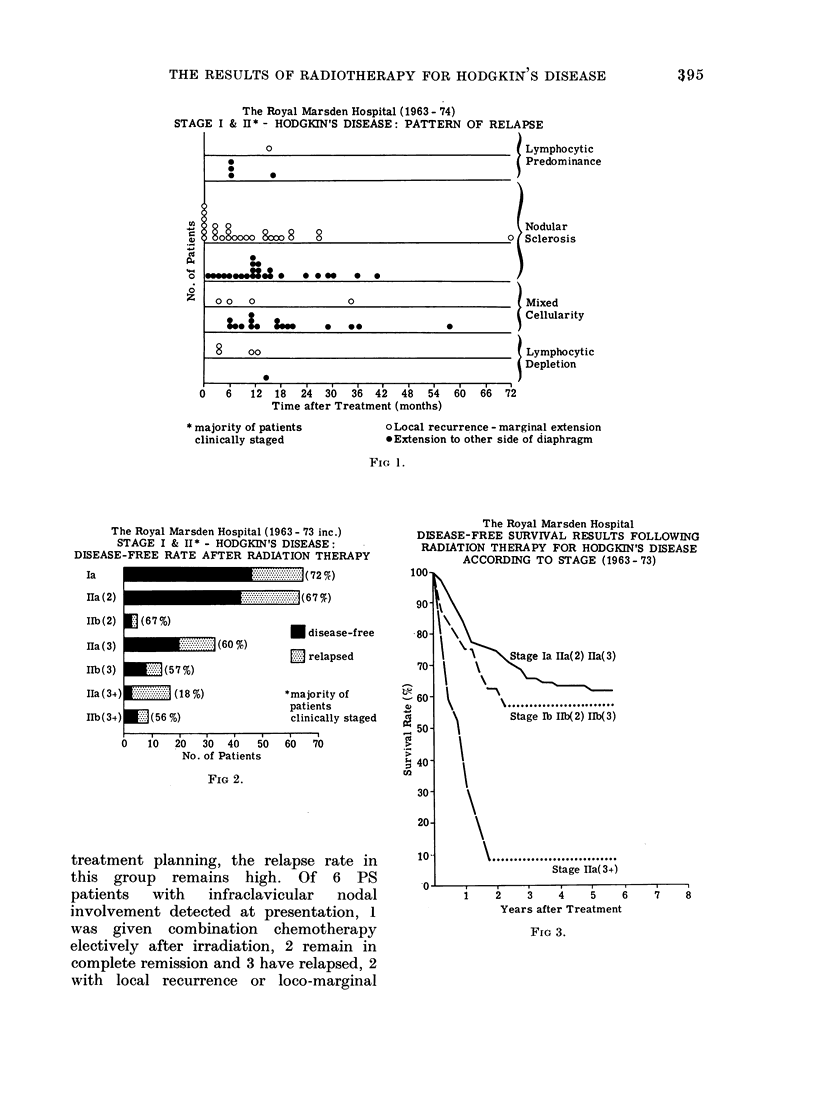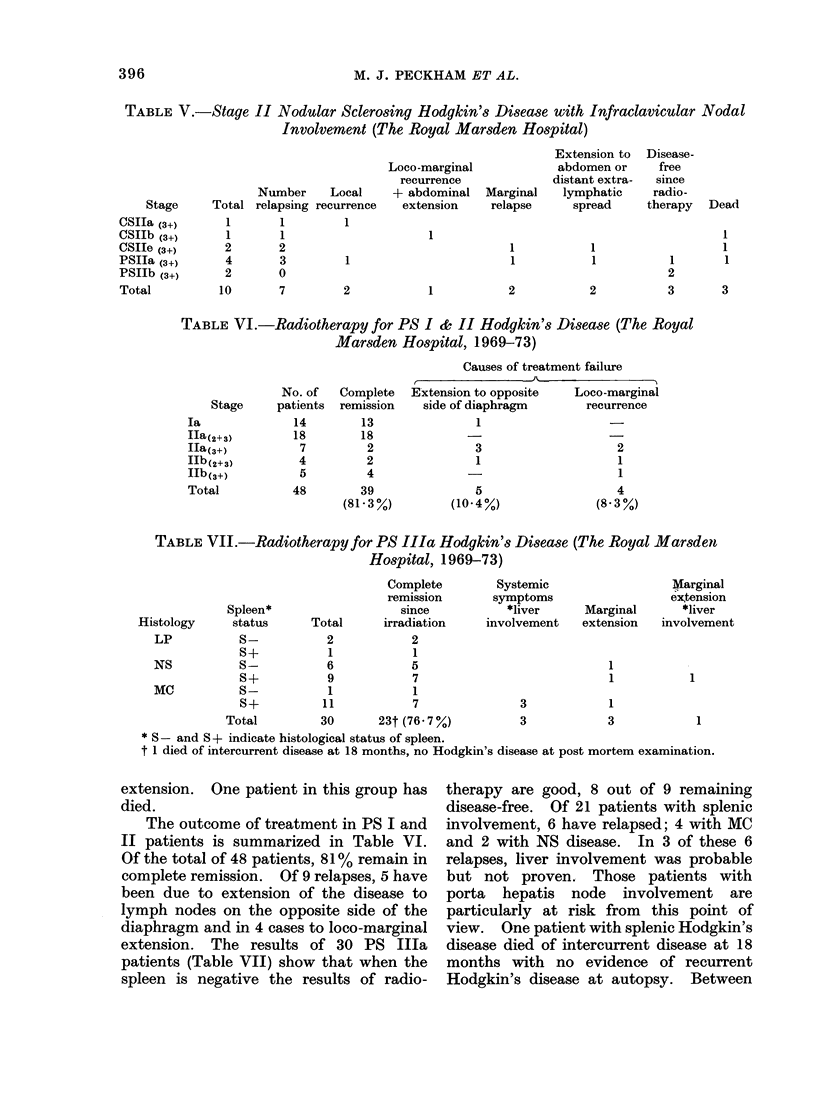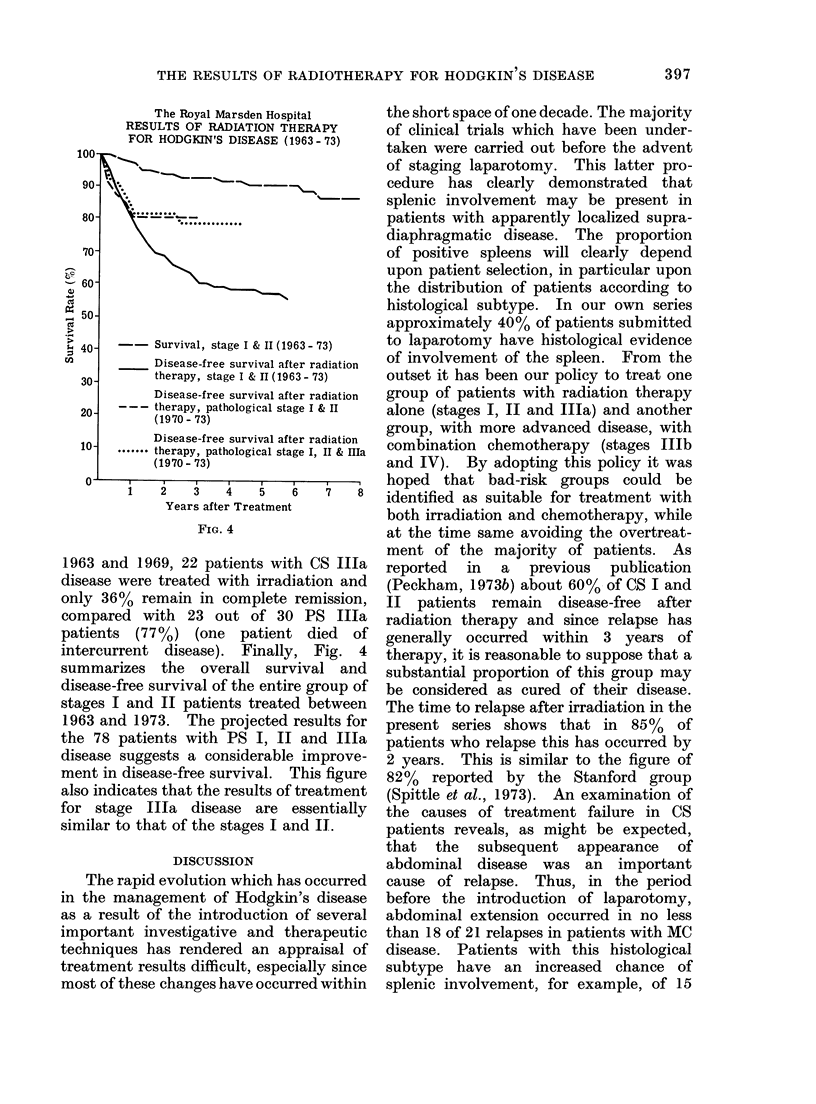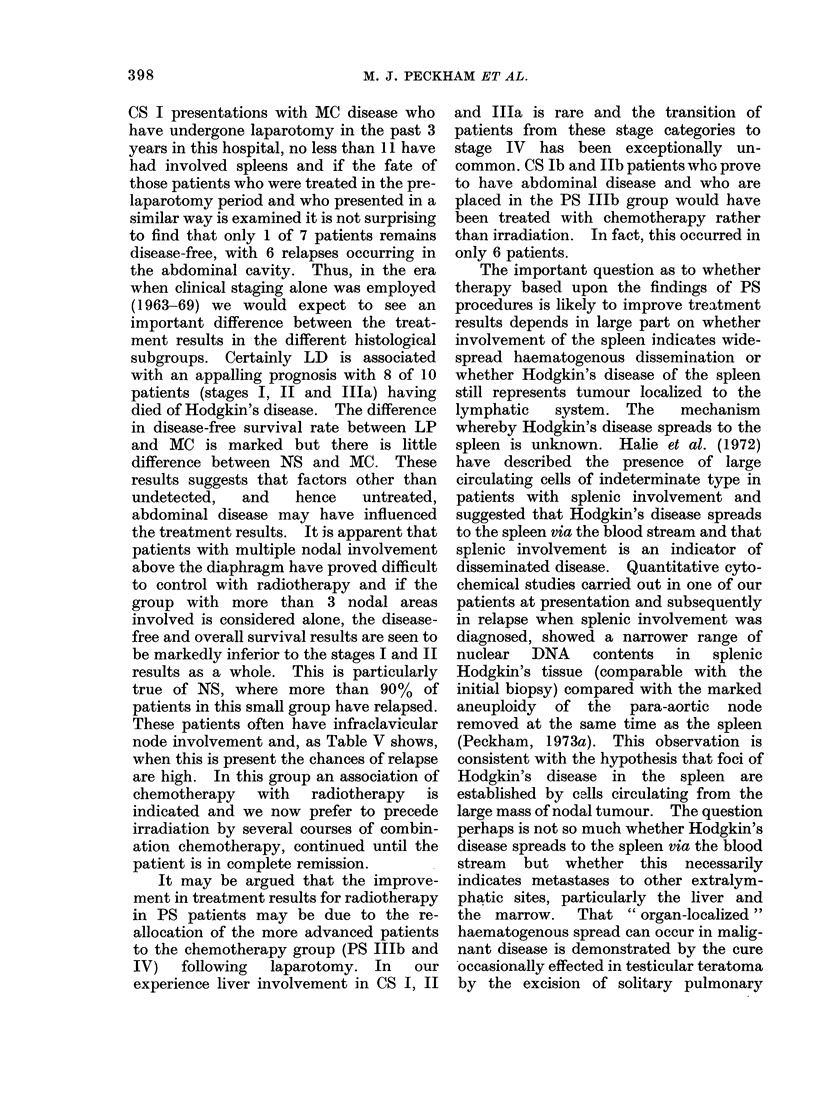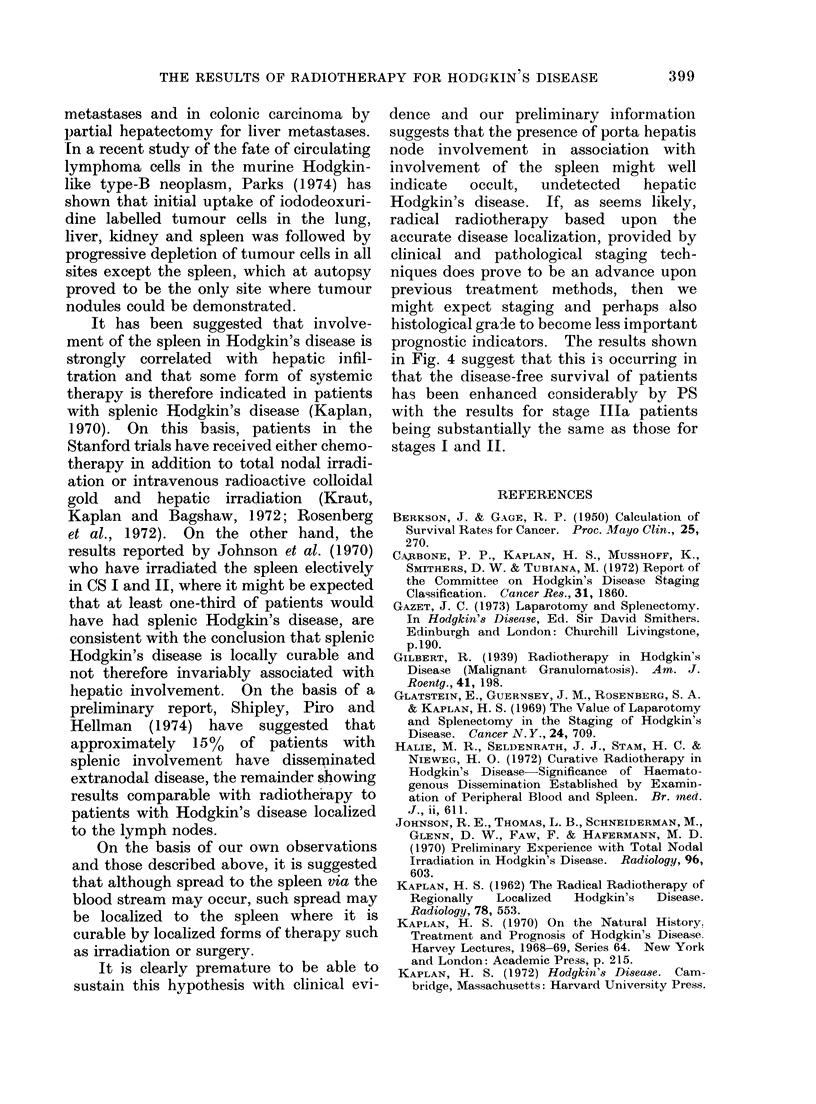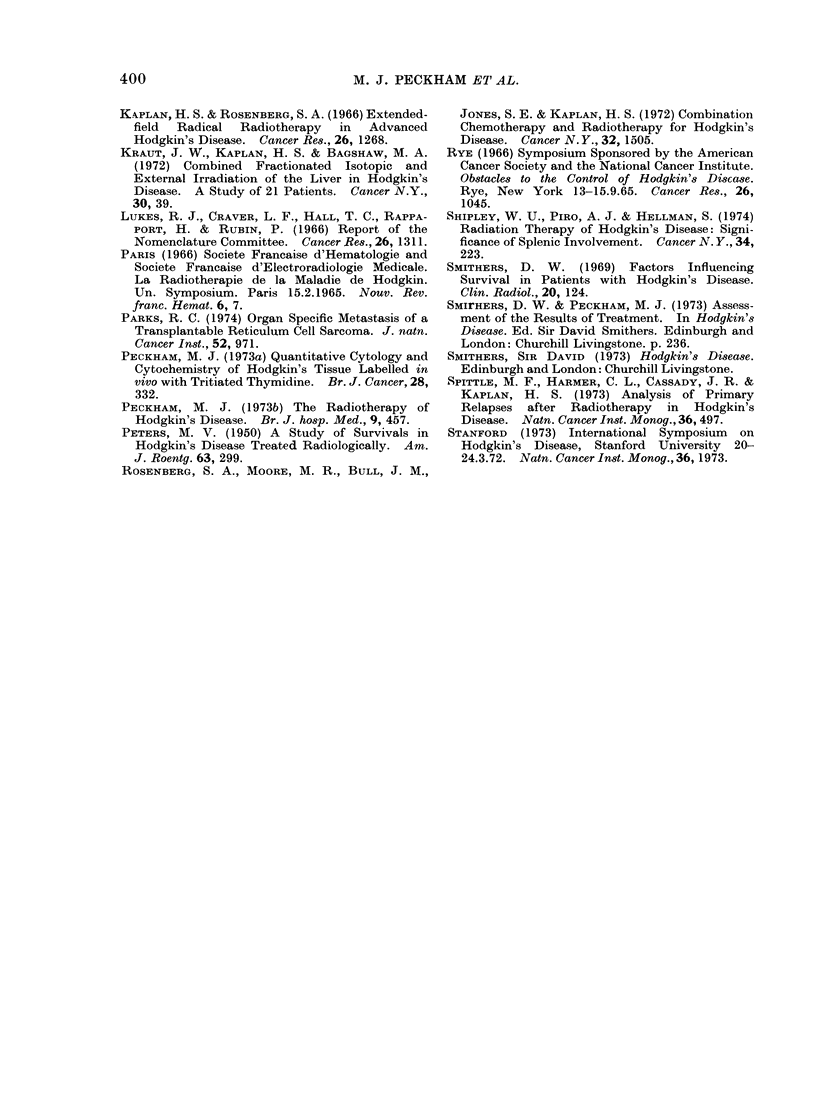# The results of radiotherapy for Hodgkins' disease.

**DOI:** 10.1038/bjc.1975.239

**Published:** 1975-09

**Authors:** M. J. Peckham, H. T. Ford, T. J. McElwain, C. L. Harmer, K. Atkinson, D. E. Austin

## Abstract

The results of radiation therapy in 212 patients with stages I and II Hodgkin's disease treated between 1963 and 1973 show that approximately 60% remain disease-free following treatment. Multiple node involvement in stage II, particularly associated with infraclavicular node disease, is identified as a group where the relapse rate is high. This presentation is associated particularly with NS. In a group of 78 patients treated with radiotherapy following staging laparotomy and splenectomy approximately 80% remain in complete remission. The preliminary results of treatment in PS IIIa patients are substantially the same as those for PS I and II; the results of treatment for NS and MC disease are similar. The significance of involvement of the spleen is discussed. Although it is probable that Hodgkin's disease spreads to the spleen through the blood stream it is suggested that splenic involvement does not necessarily indicate that the involvement of other extralymphatic structures such as liver and marrow has occurred. However, when the nodes in the porta hepatis are involved splenic Hodgkin's disease may well be associated with an increased risk of occult hepatic infiltration.


Br. J. Cancer (1975) **32**, 391

### THE RESULTS OF RADIOTHERAPY FOR HODGKIN'S DISEASE

M. J. PECKHAM, H. T. FORD, T. J. McELWAIN, C. L. HARMER, K. ATKINSON AND D. E. AUSTIN

*From the Lymphoma Unit, The Royal Marsden Hospital, Sutton, Surrey*

Received 25 April 1975. Accepted 16 May 1975

**Summary.—The results of radiation therapy in 212 patients with stages I and II Hodgkin's disease treated between 1963 and 1973 show that approximately 60% remain disease-free following treatment. Multiple node involvement in stage II, particularly associated with infraclavicular node disease, is identified as a group where the relapse rate is high. This presentation is associated particularly with NS.**

**In a group of 78 patients treated with radiotherapy following staging laparotomy and splenectomy approximately 80% remain in complete remission. The preliminary results of treatment in PS IIIa patients are substantially the same as those for PS I and II; the results of treatment for NS and MC disease are similar. The significance of involvement of the spleen is discussed. Although it is probable that Hodgkin's disease spreads to the spleen through the blood stream it is suggested that splenic involvement does not necessarily indicate that the involvement of other extralymphatic structures such as liver and marrow has occurred. However, when the nodes in the porta hepatis are involved splenic Hodgkin's disease may well be associated with an increased risk of occult hepatic infiltration.**

HODGKIN'S disease, untreated or inadequately treated, carries a grave prognosis. However, important advances in the investigation and treatment of this condition have occurred over the past 10–15 years and the major issues of pathological classification and clinical and pathological staging (Carbone *et al.*, 1972)* have been discussed and clarified at several international meetings held during this period (Paris, 1966; Rye, 1966; Stanford, 1973).

Results of previous treatment in this hospital have been reported in several publications (Smithers, 1969; Smithers and Peckham, 1973; Peckham, 1973 *b*). Experience in the biology, investigation and management of Hodgkin's disease at the Royal Marsden Hospital and Stanford University has been presented separately in monographs published in 1973 (Kaplan, 1972; Smithers, 1973).

Laparotomy and splenectomy was first employed at this centre in 1969 and experience has amply demonstrated that failure of treatment based upon a purely clinical assessment of the extent of disease may be due in large part to the presence of undetected occult abdominal tumour, particularly involvement of the spleen (Glatstein *et al.*, 1969; Gazet, 1973).

The current radiotherapeutic management of Hodgkin's disease has developed from the concepts described by Gilbert (1939) and developed by Peters (1950). An important feature of this approach is the concept of treating apparently uninvolved nodal areas adjacent to clinically obvious disease. The present form of treatment was developed by Kaplan (1962) who demonstrated that irradiation of the axial lymphatic system (total nodal irradiation) was a practical procedure (Kaplan and Rosenberg, 1966). An

* Staging according to the Ann Arbor system (Carbone *et al.*, 1972) in stage II the number of nodal areas involved is indicated as follows: $II_{(2)}$ 2 nodal areas, $II_{(3)}$ 3 nodal areas, $II_{(3+)}$ more than 3 nodal areas.

alternative approach to therapy has been advocated by Johnson *et al.* (1970) who, rather than performing a staging laparotomy, electively irradiate the spleen in clinically staged patients.

It is not proposed in this communication to review previous experience but rather to present recent results of treatment with radical radiotherapy in patients staged by clinical procedures and more recently by pathological staging techniques. Those groups of patients most at risk from relapse are identified and our preliminary experience of treatment in patients who have undergone splenectomy as part of their initial assessment is presented.

### PATIENTS AND METHODS

*Treatment groups.*—It has been our policy to reserve chemotherapy for stage IIIb and IV patients and to irradiate patients with stages I (a and b), II (a and b) and IIIa disease. This policy was adopted in order to provide a better appreciation of the causes of treatment failure and in order to identify patients in whom a combined approach would be more likely to succeed. In addition, we have been anxious to avoid overtreating patients by combining irradiation and combination chemotherapy until we were in a position to identify those groups with a high risk of relapse from either treatment used alone.

*Staging procedures.*—(Throughout this article clinical stage is abbreviated to CS and pathological stage to PS.) Clinical staging includes lymphography, intravenous pyelogram and chest x-ray, liver and spleen scans, liver function tests and bone marrow sampling (initially aspirate but since 1969 trephine, or open iliac crest biopsy). In some cases percutaneous needle biopsy of the liver was also performed. Over the past 5 years staging laparotomy has been carried out in the majority of patients at presentation. The latter procedure is now routinely carried out in clinically staged (CS) I (a and b), II (a and b) patients and stage IIIa patients. More recently, CS IIIb patients have been pathologically staged (PS) but this was not the case during the time covered by the present analysis. In patients presenting with infradiaphragmatic disease, the left scalene nodes are sampled routinely.

*Treatment methods.*—These have been described in a previous publication (Peckham, 1973*b*) and will be mentioned only briefly. Between 1963 and 1969 patients with supradiaphragmatic stage I and II disease received irradiation by the mantle technique which involves the *en-bloc* irradiation of the mediastinal, axillary and cervical nodal areas using large anterior and posterior fields, the majority of patients being treated with a linear accelerator. During this time, CS IIIa patients who did not have splenic enlargement either clinically or on scanning and in whom systemic symptoms were absent received total nodal irradiation in which the mantle treatment was followed after an interval of one month by irradiation of the para-aortic, iliac and inguinal nodes (inverted " Y " field). The spleen was not irradiated or removed in this group of patients. Patients with infradiaphragmatic stage I and II disease were treated with the inverted " Y " technique. After the introduction of laparotomy, PS I and II patients with supradiaphragmatic disease were treated initially with a mantle field but when it became evident that a small proportion were relapsing in the para-aortic region, even when both lymphogram and laparotomy were negative, it was decided to irradiate this region. This is done with a separate field extending down to L5/S1 interspace and treatment is started about one month after completion of supradiaphragmatic irradiation. PS IIIa patients receive total nodal irradiation which includes the splenic axis if the spleen was involved by tumour.

The survival rates were computed by the actuarial method incorporating a correction for expected deaths due to causes other than Hodgkin's disease. The correction was made using national mortality statistics by the method of Berkson and Gage (1950).

*Patients.*—Between 1963 and 1973, 238 stage I and II patients were treated at the Royal Marsden Hospital. Patients who were previously treated or who were lost to follow-up have been excluded leaving a total of 212 for analysis. Since the introduction of laparotomy, 78 PS I (a and b), II (a and b) and IIIa patients are available for analysis. The survival of PS I and II patients has been compared with the survival of the entire group (CS and PS) of patients treated between 1963 and 1973. The results of treatment of PS patients have not been compared with CS patients since initially many CS Ia and IIa patients did not undergo laparotomy, which was restricted to those patients considered particularly at risk.

*Patients who as a result of the findings of staging laparotomy were treated with chemotherapy rather than radiotherapy.*—Any observed improvement in the results of radiotherapy might be attributable in the splenectomy group to the re-allocation of a proportion of patients to the chemotherapy group. The only ways in which this could occur were the re-staging of CS I and IIb as PS IIIb or of any CS I and II or IIIa patients as PS stage IV. The latter transition was exceptional. During the period 1970 to 1973 21 CS Ib and IIb patients were seen and their final staging is summarized below:

| Total CS Ib, IIb | CS (No laparotomy) | PS Ib, IIb | PS IIIb |
|---|---|---|---|
| 21 | 5 | 10 | 6 |

Thus, there are only 6 patients in this group who before 1970 would have received radiotherapy but who were treated instead with radiotherapy plus chemotherapy or combination chemotherapy alone as a result of the laparotomy findings.

*Histological grading.*—(Throughout this article lymphocytic predominance is abbreviated to LP, nodular sclerosis to NS, mixed cellularity to MC and lymphocytic depletion to LD.) Histological grading has been carried out according to the modified Lukes classification (Lukes *et al.*, 1966) into the following 4 categories: lymphocytic predominance (LP), nodular sclerosis (NS), mixed cellularity (MC) and lymphocytic depletion (LD). Histological material for each patient has been reviewed at the Royal Marsden Hospital in most cases by Dr I. M. E. Hamlin.

### RESULTS

Table I summarizes the results of treatment in stage I and II patients treated between 1963 and 1973. Of this entire group of 212 patients, 164 were clinically staged and 48 pathologically staged and, as mentioned above, those patients undergoing laparotomy initially were selected in the sense that they were considered to be particularly at risk of harbouring occult intra-abdominal disease. Approximately 60% of the whole group remain in complete remission after radical radiotherapy with females faring better than males (70·2% compared with 56·8%).

TABLE I.—*Radiotherapy for Stage I & II* Hodgkin's Disease (The Royal Marsden Hospital,* 1963–73)

| | No. of patients | Disease-free since irradiation |
|---|---|---|
| Males | 118 | 67 (56·8%) |
| Females | 94 | 66 (70·2%) |
| Total | 212 | 133 (62·7%) |

* Majority clinically staged.

Table II shows the results in terms of histological grade, a diminishing disease-free survival rate is seen from LP, NS to MC, the differences between LP and MC being the most striking.

TABLE II.—*Hodgkin's Disease: Stages I & II* (The Royal Marsden Hospital* 1963–73 *inclusive*)

| Histology | Complete remission since irradiation | Total |
|---|---|---|
| LP | 23 (82%) | 28 |
| NS | 87 (62·1%) | 140 |
| MC | 23 (52%) | 44 |
| | 133 (62·7%) | 212 |

* The majority of these patients were clinically staged.

Lymphocytic depletion results are summarized in Table III. This is a small group consisting of only 10 cases presenting with stage I, II and IIIa disease.

TABLE III.—*Lymphocytic Depletion Hodgkin's Disease, Lymphographic Stages* I, II & III (*The Royal Marsden Hospital,* 1963–73)

| | | | | Survival time (months) | |
|---|---|---|---|---|---|
| Stage | Total | Alive | Dead | Alive | Dead |
| Ia | 2 | 0 | 2 | — | 9, 33 |
| IIa | 1 | 0 | 1 | — | 15 |
| IIb | 3 | 1 | 2 | 67 | 14, 33 |
| IIIa | 4 | 1 | 3 | 42 | 9, 8, 21 |

TABLE IV.—*Relapse Pattern in Stages I & II Hodgkin's Disease (LP, NS and MC) treated with Radiotherapy (The Royal Marsden Hospital* 1963–73)

| | | Total | Abdominal extension to L. neck in case of infra-diaphragmatic presentation: In CS patients | In PS patients | Abdominal extension and loco-marginal relapse | Loco-marginal relapse | Uncontrolled aggressive disease |
|---|---|---|---|---|---|---|---|
| (A) Lymphocytic predominance | Male | 5 | 4 | | | 1 | |
| | Female | 0 | | | | | |
| | Total | 5 | 4 | | | 1 | |
| (B) Nodular sclerosis† | Male | 31 | 13 | 2 | 4 | 6 | 6 |
| | Female | 22 | 5 | 1 | 2 | 12 | 2 |
| | Total | 53 | 18 | 3 | 6 | 18 | 8 |
| (C) Mixed cellularity | Male | 16 | 13* | 1 | 1 | | 1 |
| | Female | 5 | 2 | 1 | | 2 | |
| | Total | 21 | 15 | 2 | 1 | 2 | 1 |

† Of 53 relapses, 17 developed lung involvement, 5 infraclavicular node extension and 3 bone involvement.
* 3 infradiaphragmatic presentations extending to L. cervical nodes and in one case, L. axillary node.

Despite recent advances the prognosis for LD remains poor and 8 of 10 patients in this series are dead.

The causes of treatment failure in stages I and II (excluding LD) are summarized in Table IV. Of the NS patients abdominal extension was the cause of disease relapse in 18 of the CS patients but this has so far only been encountered in 3 of the PS group It is important to note that 18 patients had either local recurrence or marginal extension and 8 were considered to have aggressive disease which was not controlled by radiation therapy. In this latter group, local or marginal relapse was an important cause of treatment failure and in 4 out of 8 cases multiple nodal areas of involvement were present initially.

In 4 of the 5 relapses in patients with LP this was due to abdominal extension. Abdominal extension is particularly important in MC disease, accounting for 15 of the 21 relapses which have occurred. However, only 2 abdominal relapses have occurred in the PS group. There is thus a marked difference in the relapse pattern of MC compared with NS. In the latter group loco-marginal relapse is a relatively common cause of treatment failure whereas this is uncommon in MC. In LD 3 out of 5 patients with relapse have had uncontrolled locally aggressive disease.

The time interval between treatment and relapse in stages I and II patients is shown in Fig. 1. 54% of relapses occurred within 12 months and 85% within 24 months of radiation therapy with few occurring after 36 months. In 68% of patients with loco-marginal recurrence this became apparent within 12 months of treatment. There is a suggestion, as might be expected, that local relapse appears sooner than abdominal relapse. Fig. 2 summarizes the disease-free rates in stages I and II disease in histogram form and shows that in those patients with multiple nodal areas involved ($IIa_{(3+)}$) the relapse rate is high. Fortunately, the most common presentation within stage II is associated with involvement of 2 or 3 nodal areas. Fig. 3 shows the disease-free survival of stages I and II patients isolating those patients with more than 3 nodal areas involved. This group is frequently associated with infraclavicular disease and Table V summarizes the pretreatment disease extent in 10 patients with infraclavicular disease and their subsequent course. It is apparent that even in PS patients with considerable attention being paid to

FIG 1.

FIG 2.

FIG 3.

treatment planning, the relapse rate in this group remains high. Of 6 PS patients with infraclavicular nodal involvement detected at presentation, 1 was given combination chemotherapy electively after irradiation, 2 remain in complete remission and 3 have relapsed, 2 with local recurrence or loco-marginal

TABLE V.—*Stage II Nodular Sclerosing Hodgkin's Disease with Infraclavicular Nodal Involvement (The Royal Marsden Hospital)*

| Stage | Total | Number relapsing | Local recurrence | Loco-marginal recurrence + abdominal extension | Marginal relapse | Extension to abdomen or distant extra-lymphatic spread | Disease-free since radio-therapy | Dead |
|---|---|---|---|---|---|---|---|---|
| CSIIa (3+) | 1 | 1 | 1 | | | | | |
| CSIIb (3+) | 1 | 1 | | 1 | | | | 1 |
| CSIIe (3+) | 2 | 2 | | | 1 | 1 | | 1 |
| PSIIa (3+) | 4 | 3 | 1 | | 1 | 1 | 1 | 1 |
| PSIIb (3+) | 2 | 0 | | | | | 2 | |
| Total | 10 | 7 | 2 | 1 | 2 | 2 | 3 | 3 |

TABLE VI.—*Radiotherapy for PS I & II Hodgkin's Disease (The Royal Marsden Hospital,* 1969–73)

| Stage | No. of patients | Complete remission | Causes of treatment failure: Extension to opposite side of diaphragm | Causes of treatment failure: Loco-marginal recurrence |
|---|---|---|---|---|
| Ia | 14 | 13 | 1 | — |
| IIa(2+3) | 18 | 18 | — | — |
| IIa(3+) | 7 | 2 | 3 | 2 |
| IIb(2+3) | 4 | 2 | 1 | 1 |
| IIb(3+) | 5 | 4 | — | 1 |
| Total | 48 | 39 (81·3%) | 5 (10·4%) | 4 (8·3%) |

TABLE VII.—*Radiotherapy for PS IIIa Hodgkin's Disease (The Royal Marsden Hospital,* 1969–73)

| Histology | Spleen* status | Total | Complete remission since irradiation | Systemic symptoms *liver involvement | Marginal extension | Marginal extension *liver involvement |
|---|---|---|---|---|---|---|
| LP | S− | 2 | 2 | | | |
| | S+ | 1 | 1 | | | |
| NS | S− | 6 | 5 | | 1 | |
| | S+ | 9 | 7 | | 1 | 1 |
| MC | S− | 1 | 1 | | | |
| | S+ | 11 | 7 | 3 | 1 | |
| | Total | 30 | 23† (76·7%) | 3 | 3 | 1 |

* S− and S+ indicate histological status of spleen.
† 1 died of intercurrent disease at 18 months, no Hodgkin's disease at post mortem examination.

extension. One patient in this group has died.

The outcome of treatment in PS I and II patients is summarized in Table VI. Of the total of 48 patients, 81% remain in complete remission. Of 9 relapses, 5 have been due to extension of the disease to lymph nodes on the opposite side of the diaphragm and in 4 cases to loco-marginal extension. The results of 30 PS IIIa patients (Table VII) show that when the spleen is negative the results of radiotherapy are good, 8 out of 9 remaining disease-free. Of 21 patients with splenic involvement, 6 have relapsed; 4 with MC and 2 with NS disease. In 3 of these 6 relapses, liver involvement was probable but not proven. Those patients with porta hepatis node involvement are particularly at risk from this point of view. One patient with splenic Hodgkin's disease died of intercurrent disease at 18 months with no evidence of recurrent Hodgkin's disease at autopsy. Between

FIG. 4

1963 and 1969, 22 patients with CS IIIa disease were treated with irradiation and only 36% remain in complete remission, compared with 23 out of 30 PS IIIa patients (77%) (one patient died of intercurrent disease). Finally, Fig. 4 summarizes the overall survival and disease-free survival of the entire group of stages I and II patients treated between 1963 and 1973. The projected results for the 78 patients with PS I, II and IIIa disease suggests a considerable improvement in disease-free survival. This figure also indicates that the results of treatment for stage IIIa disease are essentially similar to that of the stages I and II.

### DISCUSSION

The rapid evolution which has occurred in the management of Hodgkin's disease as a result of the introduction of several important investigative and therapeutic techniques has rendered an appraisal of treatment results difficult, especially since most of these changes have occurred within the short space of one decade. The majority of clinical trials which have been undertaken were carried out before the advent of staging laparotomy. This latter procedure has clearly demonstrated that splenic involvement may be present in patients with apparently localized supradiaphragmatic disease. The proportion of positive spleens will clearly depend upon patient selection, in particular upon the distribution of patients according to histological subtype. In our own series approximately 40% of patients submitted to laparotomy have histological evidence of involvement of the spleen. From the outset it has been our policy to treat one group of patients with radiation therapy alone (stages I, II and IIIa) and another group, with more advanced disease, with combination chemotherapy (stages IIIb and IV). By adopting this policy it was hoped that bad-risk groups could be identified as suitable for treatment with both irradiation and chemotherapy, while at the time same avoiding the overtreatment of the majority of patients. As reported in a previous publication (Peckham, 1973*b*) about 60% of CS I and II patients remain disease-free after radiation therapy and since relapse has generally occurred within 3 years of therapy, it is reasonable to suppose that a substantial proportion of this group may be considered as cured of their disease. The time to relapse after irradiation in the present series shows that in 85% of patients who relapse this has occurred by 2 years. This is similar to the figure of 82% reported by the Stanford group (Spittle *et al.*, 1973). An examination of the causes of treatment failure in CS patients reveals, as might be expected, that the subsequent appearance of abdominal disease was an important cause of relapse. Thus, in the period before the introduction of laparotomy, abdominal extension occurred in no less than 18 of 21 relapses in patients with MC disease. Patients with this histological subtype have an increased chance of splenic involvement, for example, of 15

CS I presentations with MC disease who have undergone laparotomy in the past 3 years in this hospital, no less than 11 have had involved spleens and if the fate of those patients who were treated in the pre-laparotomy period and who presented in a similar way is examined it is not surprising to find that only 1 of 7 patients remains disease-free, with 6 relapses occurring in the abdominal cavity. Thus, in the era when clinical staging alone was employed (1963–69) we would expect to see an important difference between the treatment results in the different histological subgroups. Certainly LD is associated with an appalling prognosis with 8 of 10 patients (stages I, II and IIIa) having died of Hodgkin's disease. The difference in disease-free survival rate between LP and MC is marked but there is little difference between NS and MC. These results suggests that factors other than undetected, and hence untreated, abdominal disease may have influenced the treatment results. It is apparent that patients with multiple nodal involvement above the diaphragm have proved difficult to control with radiotherapy and if the group with more than 3 nodal areas involved is considered alone, the disease-free and overall survival results are seen to be markedly inferior to the stages I and II results as a whole. This is particularly true of NS, where more than 90% of patients in this small group have relapsed. These patients often have infraclavicular node involvement and, as Table V shows, when this is present the chances of relapse are high. In this group an association of chemotherapy with radiotherapy is indicated and we now prefer to precede irradiation by several courses of combination chemotherapy, continued until the patient is in complete remission.

It may be argued that the improvement in treatment results for radiotherapy in PS patients may be due to the reallocation of the more advanced patients to the chemotherapy group (PS IIIb and IV) following laparotomy. In our experience liver involvement in CS I, II and IIIa is rare and the transition of patients from these stage categories to stage IV has been exceptionally uncommon. CS Ib and IIb patients who prove to have abdominal disease and who are placed in the PS IIIb group would have been treated with chemotherapy rather than irradiation. In fact, this occurred in only 6 patients.

The important question as to whether therapy based upon the findings of PS procedures is likely to improve treatment results depends in large part on whether involvement of the spleen indicates widespread haematogenous dissemination or whether Hodgkin's disease of the spleen still represents tumour localized to the lymphatic system. The mechanism whereby Hodgkin's disease spreads to the spleen is unknown. Halie *et al.* (1972) have described the presence of large circulating cells of indeterminate type in patients with splenic involvement and suggested that Hodgkin's disease spreads to the spleen *via* the blood stream and that splenic involvement is an indicator of disseminated disease. Quantitative cytochemical studies carried out in one of our patients at presentation and subsequently in relapse when splenic involvement was diagnosed, showed a narrower range of nuclear DNA contents in splenic Hodgkin's tissue (comparable with the initial biopsy) compared with the marked aneuploidy of the para-aortic node removed at the same time as the spleen (Peckham, 1973*a*). This observation is consistent with the hypothesis that foci of Hodgkin's disease in the spleen are established by cells circulating from the large mass of nodal tumour. The question perhaps is not so much whether Hodgkin's disease spreads to the spleen *via* the blood stream but whether this necessarily indicates metastases to other extralymphatic sites, particularly the liver and the marrow. That "organ-localized" haematogenous spread can occur in malignant disease is demonstrated by the cure occasionally effected in testicular teratoma by the excision of solitary pulmonary metastases and in colonic carcinoma by partial hepatectomy for liver metastases. In a recent study of the fate of circulating lymphoma cells in the murine Hodgkin-like type-B neoplasm, Parks (1974) has shown that initial uptake of iododeoxuridine labelled tumour cells in the lung, liver, kidney and spleen was followed by progressive depletion of tumour cells in all sites except the spleen, which at autopsy proved to be the only site where tumour nodules could be demonstrated.

It has been suggested that involvement of the spleen in Hodgkin's disease is strongly correlated with hepatic infiltration and that some form of systemic therapy is therefore indicated in patients with splenic Hodgkin's disease (Kaplan, 1970). On this basis, patients in the Stanford trials have received either chemotherapy in addition to total nodal irradiation or intravenous radioactive colloidal gold and hepatic irradiation (Kraut, Kaplan and Bagshaw, 1972; Rosenberg *et al.*, 1972). On the other hand, the results reported by Johnson *et al.* (1970) who have irradiated the spleen electively in CS I and II, where it might be expected that at least one-third of patients would have had splenic Hodgkin's disease, are consistent with the conclusion that splenic Hodgkin's disease is locally curable and not therefore invariably associated with hepatic involvement. On the basis of a preliminary report, Shipley, Piro and Hellman (1974) have suggested that approximately 15% of patients with splenic involvement have disseminated extranodal disease, the remainder showing results comparable with radiotherapy to patients with Hodgkin's disease localized to the lymph nodes.

On the basis of our own observations and those described above, it is suggested that although spread to the spleen *via* the blood stream may occur, such spread may be localized to the spleen where it is curable by localized forms of therapy such as irradiation or surgery.

It is clearly premature to be able to sustain this hypothesis with clinical evidence and our preliminary information suggests that the presence of porta hepatis node involvement in association with involvement of the spleen might well indicate occult, undetected hepatic Hodgkin's disease. If, as seems likely, radical radiotherapy based upon the accurate disease localization, provided by clinical and pathological staging techniques does prove to be an advance upon previous treatment methods, then we might expect staging and perhaps also histological grade to become less important prognostic indicators. The results shown in Fig. 4 suggest that this is occurring in that the disease-free survival of patients has been enhanced considerably by PS with the results for stage IIIa patients being substantially the same as those for stages I and II.

### REFERENCES

BERKSON, J. & GAGE, R. P. (1950) Calculation of Survival Rates for Cancer. *Proc. Mayo Clin.*, **25**, 270.

CARBONE, P. P., KAPLAN, H. S., MUSSHOFF, K., SMITHERS, D. W. & TUBIANA, M. (1972) Report of the Committee on Hodgkin's Disease Staging Classification. *Cancer Res.*, **31**, 1860.

GAZET, J. C. (1973) Laparotomy and Splenectomy. In *Hodgkin's Disease*, Ed. Sir David Smithers. Edinburgh and London: Churchill Livingstone, p.190.

GILBERT, R. (1939) Radiotherapy in Hodgkin's Disease (Malignant Granulomatosis). *Am. J. Roentg.*, **41**, 198.

GLATSTEIN, E., GUERNSEY, J. M., ROSENBERG, S. A. & KAPLAN, H. S. (1969) The Value of Laparotomy and Splenectomy in the Staging of Hodgkin's Disease. *Cancer N.Y.*, **24**, 709.

HALIE, M. R., SELDENRATH, J. J., STAM, H. C. & NIEWEG, H. O. (1972) Curative Radiotherapy in Hodgkin's Disease—Significance of Haematogenous Dissemination Established by Examination of Peripheral Blood and Spleen. *Br. med. J.*, ii, 611.

JOHNSON, R. E., THOMAS, L. B., SCHNEIDERMAN, M., GLENN, D. W., FAW, F. & HAFERMANN, M. D. (1970) Preliminary Experience with Total Nodal Irradiation in Hodgkin's Disease. *Radiology*, **96**, 603.

KAPLAN, H. S. (1962) The Radical Radiotherapy of Regionally Localized Hodgkin's Disease. *Radiology*, **78**, 553.

KAPLAN, H. S. (1970) On the Natural History, Treatment and Prognosis of Hodgkin's Disease. Harvey Lectures, 1968–69, Series 64. New York and London: Academic Press, p. 215.

KAPLAN, H. S. (1972) *Hodgkin's Disease*. Cambridge, Massachusetts: Harvard University Press.

KAPLAN, H. S. & ROSENBERG, S. A. (1966) Extended-field Radical Radiotherapy in Advanced Hodgkin's Disease. *Cancer Res.*, **26**, 1268.

KRAUT, J. W., KAPLAN, H. S. & BAGSHAW, M. A. (1972) Combined Fractionated Isotopic and External Irradiation of the Liver in Hodgkin's Disease. A Study of 21 Patients. *Cancer N.*Y., **30**, 39.

LUKES, R. J., CRAVER, L. F., HALL, T. C., RAPPAPORT, H. & RUBIN, P. (1966) Report of the Nomenclature Committee. *Cancer Res.*, **26**, 1311.

PARIS (1966) Societe Francaise d'Hematologie and Societe Francaise d'Electroradiologie Medicale. La Radiotherapie de la Maladie de Hodgkin. Un. Symposium. Paris 15.2.1965. *Nouv. Rev. franc. Hemat.* **6**, 7.

PARKS, R. C. (1974) Organ Specific Metastasis of a Transplantable Reticulum Cell Sarcoma. *J. natn. Cancer Inst.*, **52**, 971.

PECKHAM, M. J. (1973*a*) Quantitative Cytology and Cytochemistry of Hodgkin's Tissue Labelled *in vivo* with Tritiated Thymidine. *Br. J. Cancer*, **28**, 332.

PECKHAM, M. J. (1973*b*) The Radiotherapy of Hodgkin's Disease. *Br. J. hosp. Med.*, **9**, 457.

PETERS, M. V. (1950) A Study of Survivals in Hodgkin's Disease Treated Radiologically. *Am. J. Roentg.* **63**, 299.

ROSENBERG, S. A., MOORE, M. R., BULL, J. M., JONES, S. E. & KAPLAN, H. S. (1972) Combination Chemotherapy and Radiotherapy for Hodgkin's Disease. *Cancer N.Y.*, **32**, 1505.

RYE (1966) Symposium Sponsored by the American Cancer Society and the National Cancer Institute. *Obstacles to the Control of Hodgkin's Disease.* Rye, New York 13–15.9.65. *Cancer Res.*, **26**, 1045.

SHIPLEY, W. U., PIRO, A. J. & HELLMAN, S. (1974) Radiation Therapy of Hodgkin's Disease: Significance of Splenic Involvement. *Cancer N.Y.*, **34**, 223.

SMITHERS, D. W. (1969) Factors Influencing Survival in Patients with Hodgkin's Disease. *Clin. Radiol.*, **20**, 124.

SMIrHERS, D. W. & PECKHAM, M. J. (1973) Assessment of the Results of Treatment. In *Hodgkin's Disease*. Ed. Sir David Smithers. Edinburgh and London: Churchill Livingstone. p. 236.

SMITHERS, SIR DAVID (1973) *Hodgkin's Disease.* Edinburgh and London: Churchill Livingstone.

SPITTLE, M. F., HARMER, C. L., CASSADY, J. R. & KAPLAN, H. S. (1973) Analysis of Primary Relapses after Radiotherapy in Hodgkin's Disease. *Natn. Cancer Inst. Monog.*, **36**, 497.

STANFORD (1973) International Symposium on Hodgkin's Disease, Stanford University 20–24.3.72. *Natn. Cancer Inst. Monog.*, **36**, 1973.